# Supplementary material for: Ultrafast isomerization-induced cooperative motions to higher molecular orientation in smectic liquid-crystalline azobenzene molecules
Source: Nat Commun. 2019 Sep 13;10:4159. doi: 10.1038/s41467-019-12116-6 (PMC6744564; doi:10.1038/s41467-019-12116-6)
Supplement: Supplementary file 1 — Supplementary Information [file 41467_2019_12116_MOESM1_ESM.pdf]

Supplementary information

“Ultrafast Isomerization-Induced Cooperative Motions to Higher Molecular Orientation in Smectic Liquid-Crystalline Azobenzene Molecules”

M. Hada et al.

# 1. Static characterization of LC azobenzene molecules

## Supplementary Note 1

Compound **1** was prepared based on a previous reported procedure[1]. $^{1}$H and $^{13}$C NMR spectra were recorded on a JEOL ECX-400. Elemental analysis was carried out on an Exeter Analytical CE440 elemental analyzer. MALDI TOF mass spectra were recorded on a Bruker Autoflex Speed TOF/TOF.

Bis(4-dodecylphenyl)diazene (**1**): $^{1}$H NMR (400 MHz, $CDCl_3$): δ 7.82 (d, *J* = 8.4 Hz, 4H), 7.32 (d, *J* = 8.4 Hz, 4H), 2.67 (t, *J* = 7.8 Hz, 4H), 1.65 (quin, *J* = 7.6 Hz, 4H), 1.36–1.22 (m, 36H), 0.88 (t, *J* = 6.8 Hz, 6H). $^{13}$C NMR (100 MHz, $CDCl_3$): δ 151.12, 146.31, 129.14, 122.79, 35.98, 32.02, 31.42, 29.75, 29.68, 29.59, 29.45, 29.38, 22.79, 14.22. Elemental analysis: Calcd. (%) for $C_{36}H_{58}N_2$: C, 83.33; H, 11.27; N, 5.40. Found: C, 83.42; H, 11.36; N, 5.68. MS (MALDI-TOF): calcd, 519.46 ($[M+H]^+$); found, 519.26.

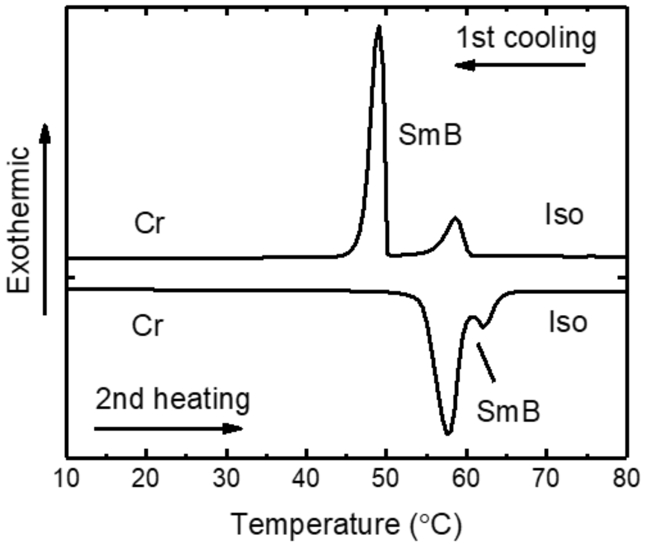


**Supplementary Figure 1**. Differential scanning calorimetry curves of the azobenzene molecules obtained at a scanning rate of 10°C min$^{-1}$. Azobenzene exhibits a crystalline phase at room temperature, a smectic B phase between 50°C to 60°C, and an isotropic phase above 60 °C. The labels Cr, SmB, and Iso inset in the figure indicate the crystalline phase, smectic B phase, and isotropic phase, respectively.

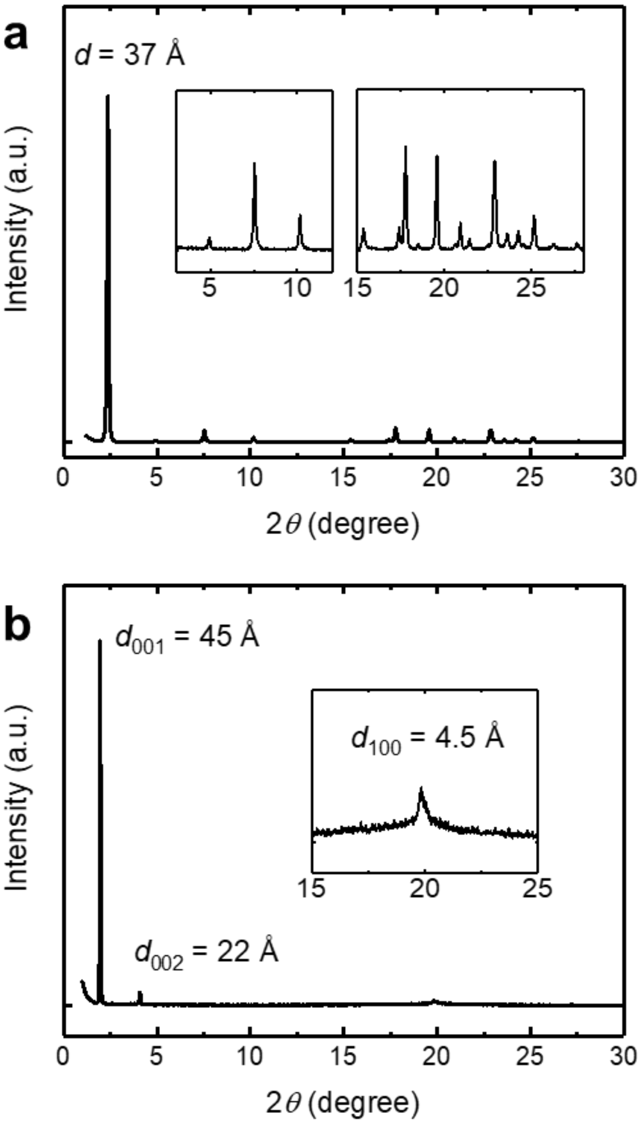


**Supplementary Figure 2.** X-ray diffraction patterns of the azobenzene molecules in the bulk state at 20°C (a) and 55°C (b) upon heating. In the liquid crystalline (LC) phase (b), the longitudinal and lateral lengths ($d_{001}$ and $d_{100}$) of the azobenzene molecule are 45 Å and 4.5 Å, respectively. Since the molecules have a six-fold symmetric structure, the intermolecular distance in the lateral direction was determined to be 5.1 Å ($= 2/\sqrt{3} \times$ 4.5 Å ).

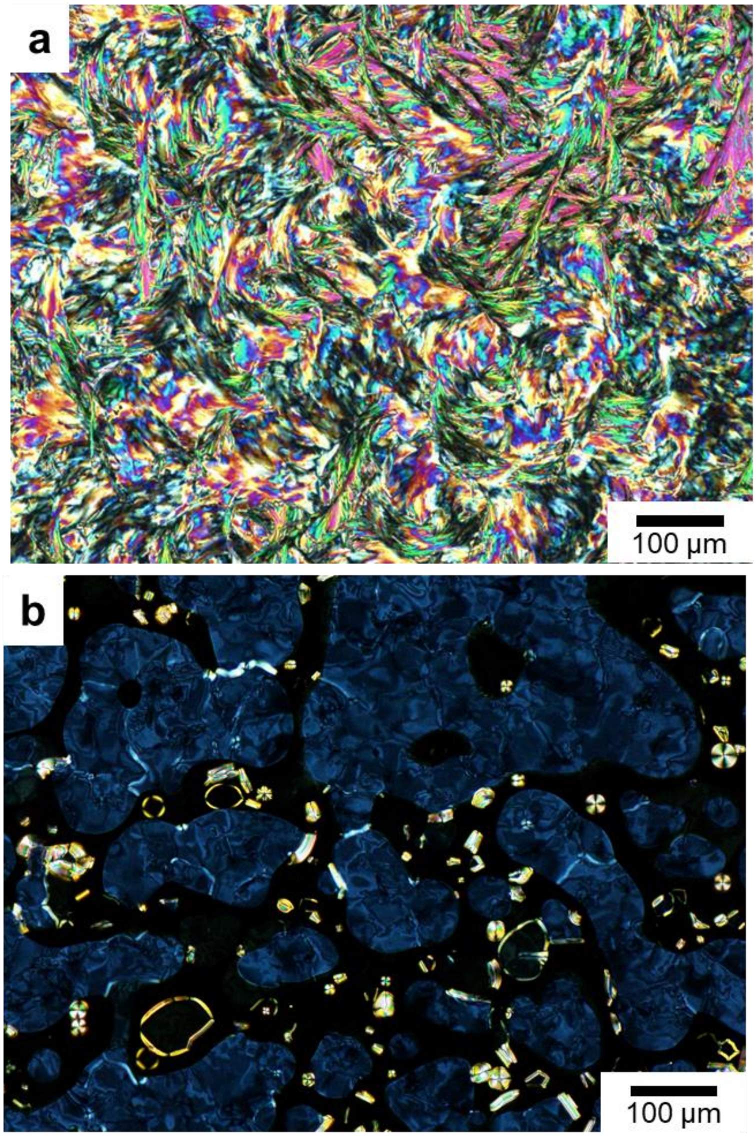


**Supplementary Figure 3.** Polarized optical microscopy images of the azobenzene molecules in a glass cell (5 μm thick) in the crystal phase at 20°C (a) and in the smectic B phase at 51°C (b).

## 2. Transient absorption of LC azobenzene molecules

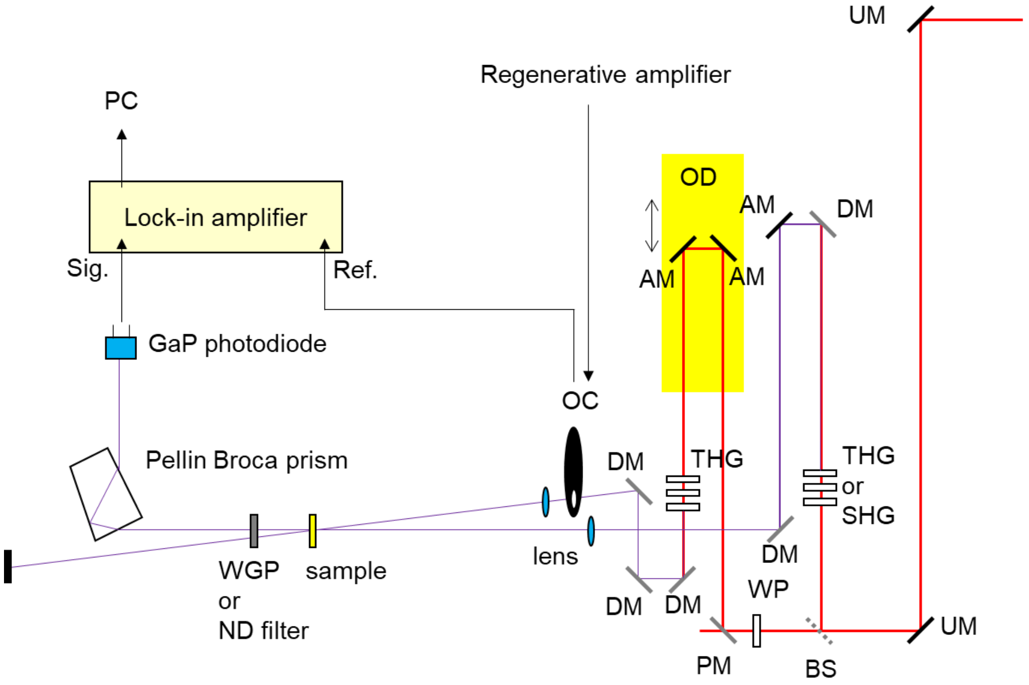


**Supplementary Figure 4.** Experimental setup for the transient transmission measurements of azobenzene in the crystalline and LC phases. The optical pulses (wavelength: 800 nm, pulse duration: 100 fs, power: 2 mJ/pulse, repetition rate: 500 Hz) generated by the regenerative amplifier (Spitfire XP, Spectra-Physics) were separated into two arms by the beam splitter (BS). The pump pulses were input into the optical delay line (OD), converted to ultraviolet (UV) light (266 nm) by β-barium borate (BBO) crystals, and changed their repetition rate to 250 Hz by passing through the optical chopper (OC) synchronized with the regenerative amplifier. The intensity of the pump-light was tuned by the waveplate (WP) and polarized mirror (PM). The probe pulses were converted to near-UV (400 nm) or UV (266 nm) light by the BBO crystals. The pump and probe pulses were polarized vertically and horizontally with respect to the optical table, respectively. The pump and probe pulses were focused onto the same spot by the lens, and the transmitted probe light was detected by the gallium phosphide (GaP) photodetector and obtained by the lock-in amplifier. To reduce the noise from the pump pulses, we subtracted the signal without probe pulses from the signal with probe pulses. To remove the scattered pump pulses, we used a wire grid polarizer (WGP) for UV light or a neutral density (ND) filter made from BK7 glass. The Pellin Broca prism was also used to remove the scattered pump light. UM, DM, and AM represent an ultrafast mirror (low group delay dispersion mirror), a dichroic mirror, and a UV-enhanced aluminium mirror, respectively.

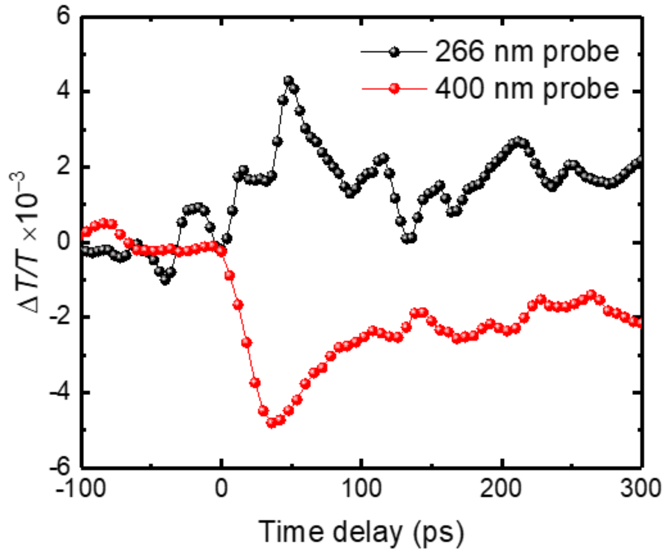


**Supplementary Figure 5.** The ultrafast transient transmission spectra of azobenzene molecules in the crystalline phase (20°C). Time zero was obtained from the transient transmission spectrum at a probe wavelength of 400 nm. In the crystalline phase, the electronic transitions and photoisomerization of the isolated molecules located at the surface and interface may be observed, since *trans*-to-*cis* isomerization should not occur in molecules under strong crystal packing.

## 3. Electron diffraction of LC azobenzene molecules

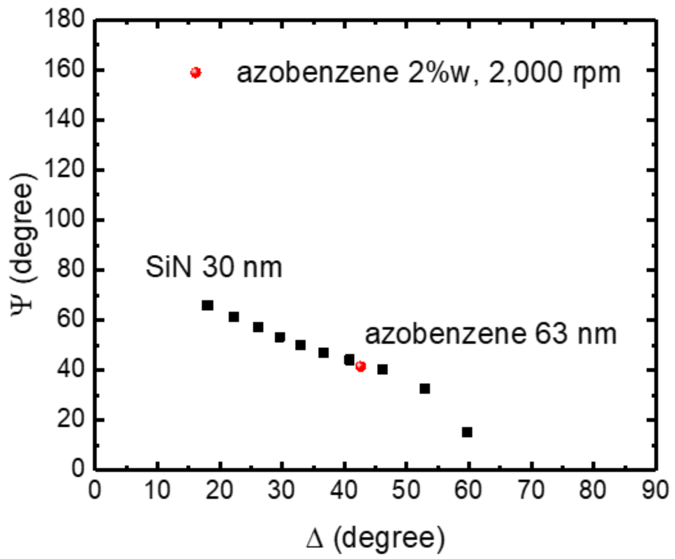


**Supplementary Figure 6.** Single-wavelength ellipsometry map of the azobenzene film[2]. The black symbols indicate film thicknesses of 0, 10, 20, … 80, and 90 nm. The wavelength of the light source was 635 nm. The thickness and refractive index of the film were determined to be ~60 nm and $n$ = 1.6, respectively.

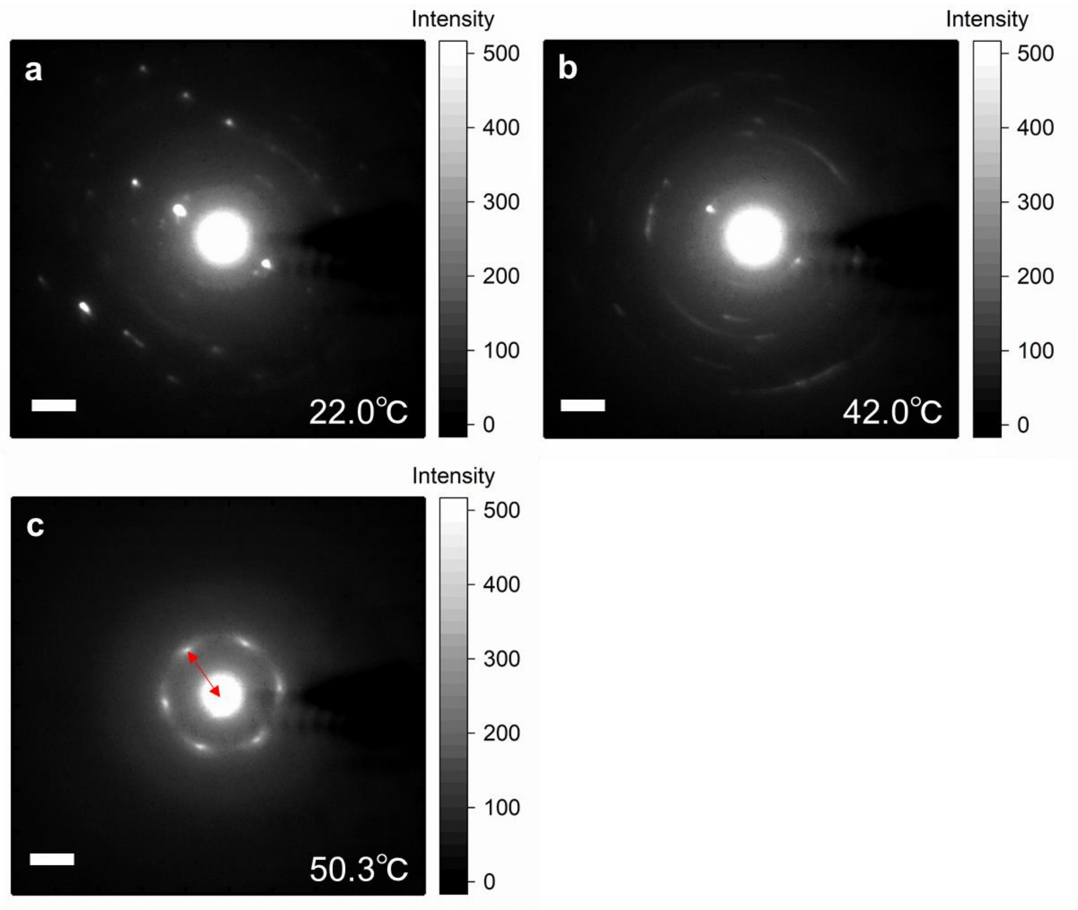


**Supplementary Figure 7.** Electron diffraction patterns from azobenzene in the crystalline phase at 22.0 °C (a), in the poly-crystalline phase at 42.0 °C (b), and in the LC phase at 50.3 °C (c). The length of the red arrow in (c) indicates the reciprocal molecular distance ($Q$ = 1.4 Å$^{-1}$, $d_{100}$ = 4.5 Å). The inset white scale bars correspond to $Q$ = 1 Å$^{-1}$.

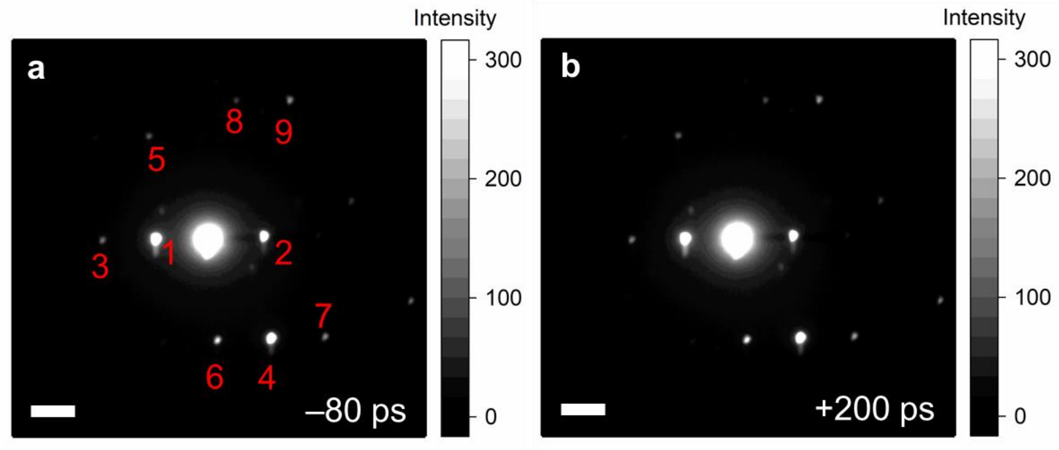


**Supplementary Figure 8.** Electron diffraction patterns from azobenzene molecules in the crystalline phase (20°C) before (a) and after (b) photoexcitation with the linearly polarized UV light at an incident fluence of 300 μJ $cm^{-2}$. The changes in the intensities of the numbered diffraction spots as functions of time are shown in the next figure. The inset white scale bars correspond to $Q = 1$ $Å^{-1}$.

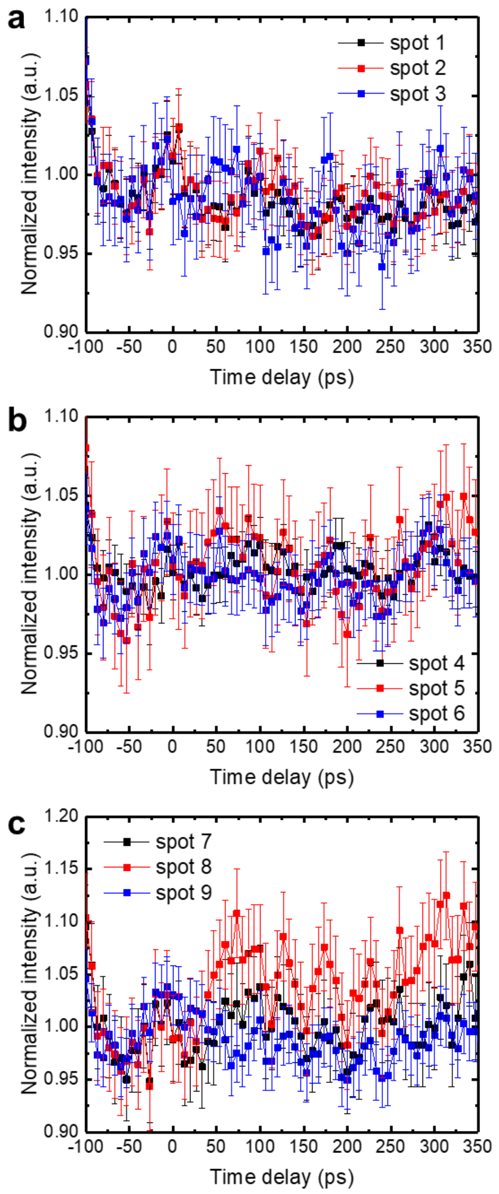


**Supplementary Figure 9.** Changes in the intensities of the spots numbered 1–3 (a), 4–6 (b), and 7–9 (c) as in Supplementary Fig. 8 as functions of time. No systematic change in intensity under UV photoexcitation was observed in the azobenzene molecules in the crystalline phase. The error bars represent the standard deviation at each time delay.

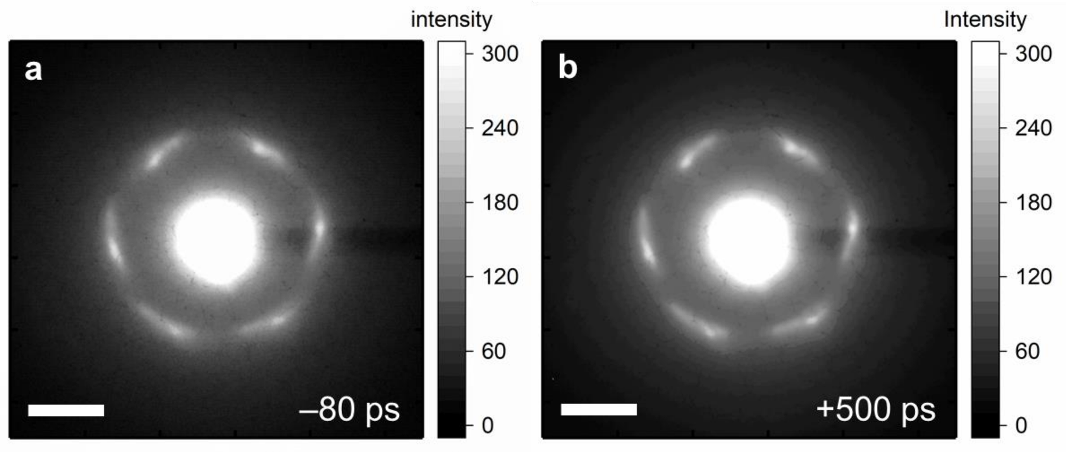


**Supplementary Figure 10.** Electron diffraction patterns of azobenzene molecules in the LC phase (53°C) before (a) and after (b) photoexcitation with linearly polarized UV light at an incident fluence of 300 μJ cm$^{-2}$. The repetition rate of the laser was fixed at 200 Hz. The inset white scale bars correspond to $Q$ = 1 Å$^{-1}$.

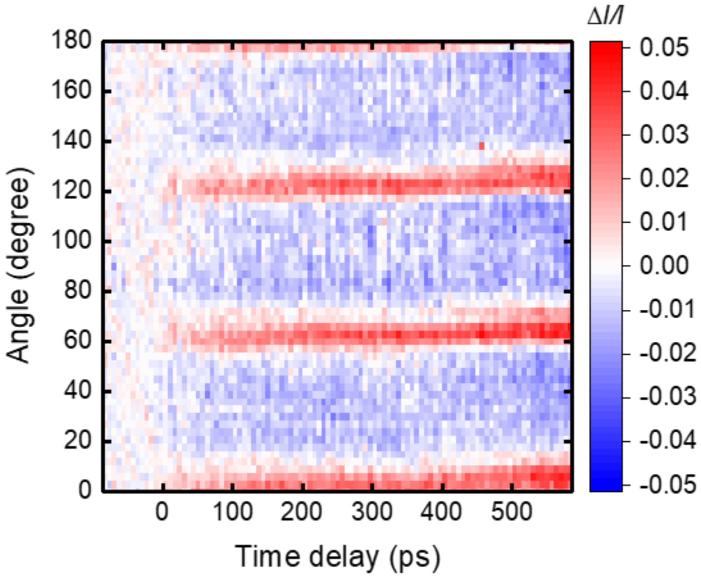


**Supplementary Figure 11.** Two-dimensional map of the angular- and time-dependent diffraction intensities at a $Q$-value (Supplementary Fig. 10) of 1.4 Å$^{-1}$. The map shows the constructive molecular ordering at angles of 5, 65, and 125°.

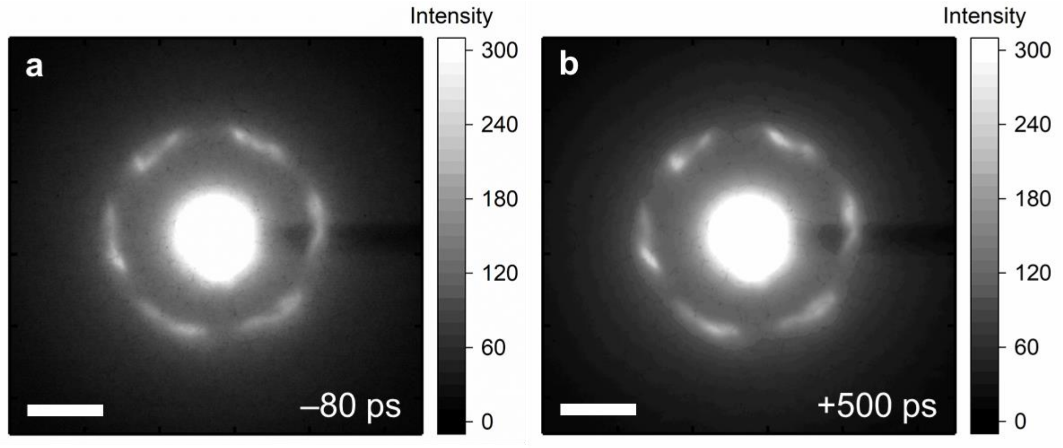


**Supplementary Figure 12.** Electron diffraction patterns of azobenzene molecules in the LC phase (53°C) before (a) and after (b) excitation with linearly polarized UV light at an incident fluence of 500 μJ $cm^{-2}$. The repetition rate of the laser was fixed at 200 Hz. The inset white scale bars correspond to $Q$ = 1 $Å^{-1}$.

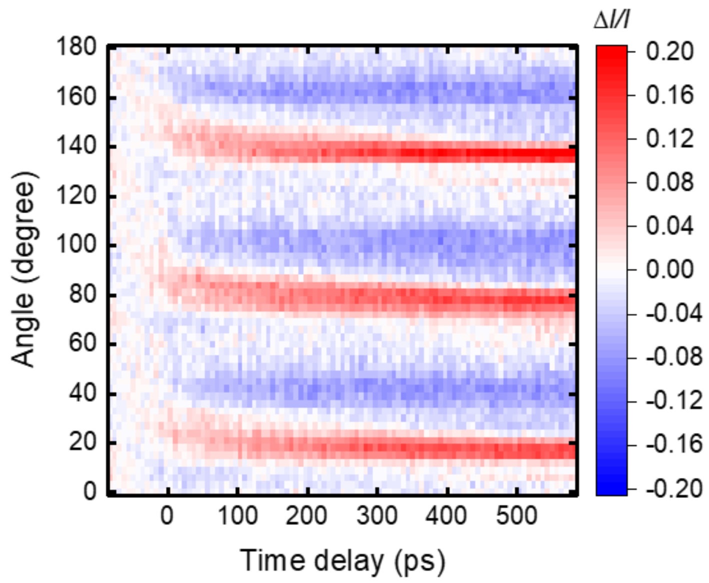


**Supplementary Figure 13.** Two-dimensional map of the angular- and time-dependent diffraction intensities at the $Q$-value (Supplementary Fig. 12) of 1.4 Å$^{-1}$. The map shows the constructive molecular ordering at angles of 15, 75, and 135°.

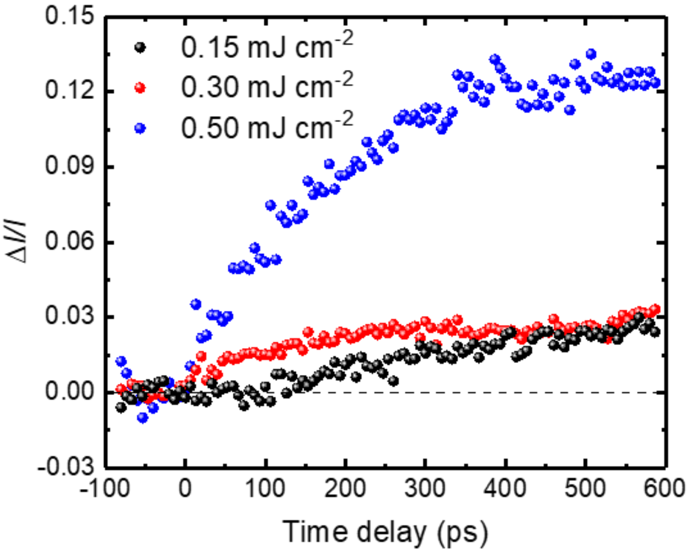


**Supplementary Figure 14.** Power dependence of the changes in molecular orientation as a function of time.

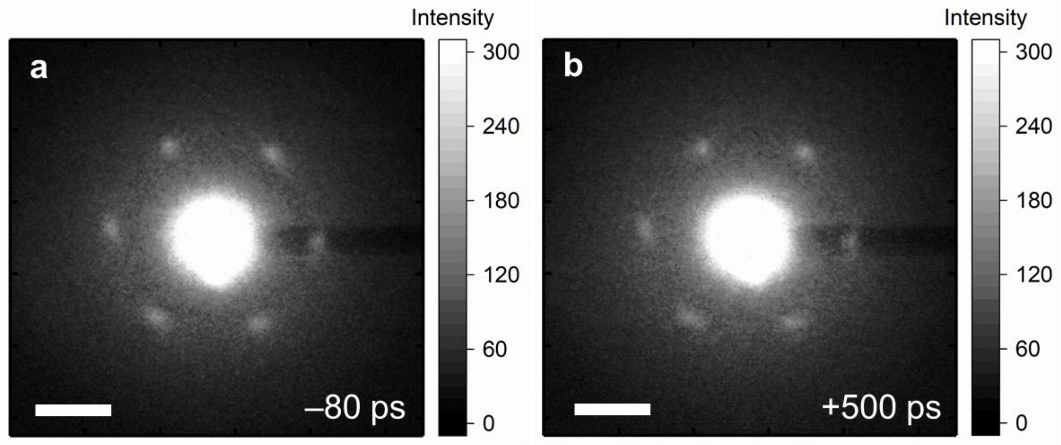


**Supplementary Figure 15.** Electron diffraction patterns of azobenzene molecules in the LC phase (53°C) before (a) and after (b) excitation with circularly polarized UV light at an incident fluence of 500 μJ $cm^{-2}$. The repetition rate of the laser was fixed at 200 Hz. The inset white scale bars correspond to $Q = 1$ $Å^{-1}$.

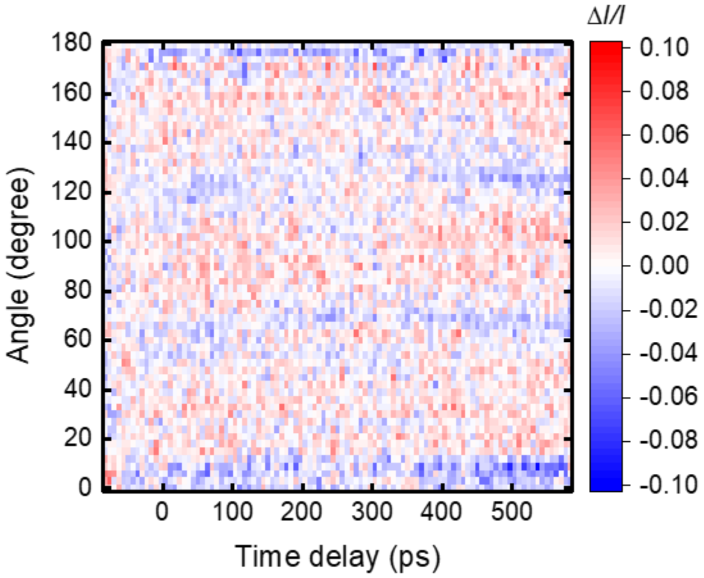


**Supplementary Figure 16.** Two-dimensional map of the angular- and time-dependent diffraction intensities (Supplementary Fig. 15) at the $Q$-value of 1.4 $Å^{-1}$. The map shows neither constructive nor destructive ordering.

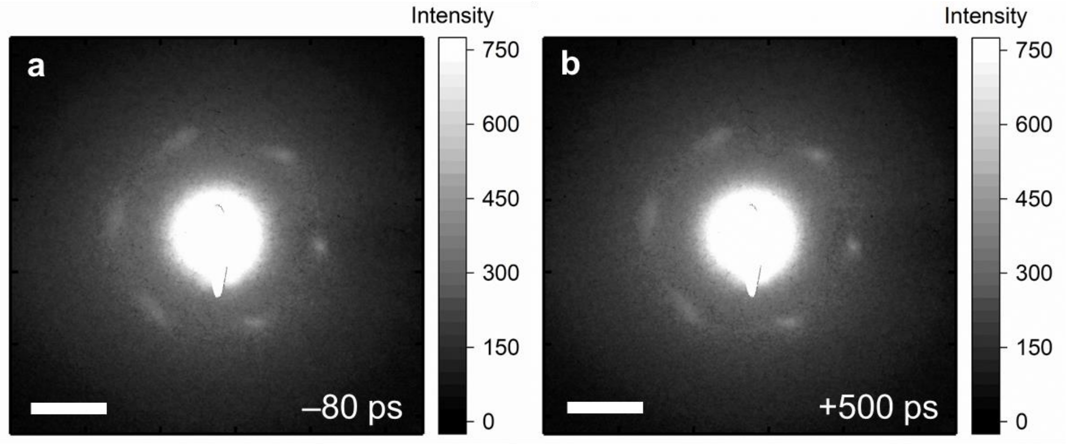


**Supplementary Figure 17.** Electron diffraction patterns of azobenzene molecules in the LC phase (53°C) before (a) and after (b) excitation with linearly polarized UV light at an incident fluence of 500 μJ cm$^{-2}$. The repetition rate of the laser was fixed at 1 kHz. The inset white scale bars correspond to $Q$ = 1 Å$^{-1}$.

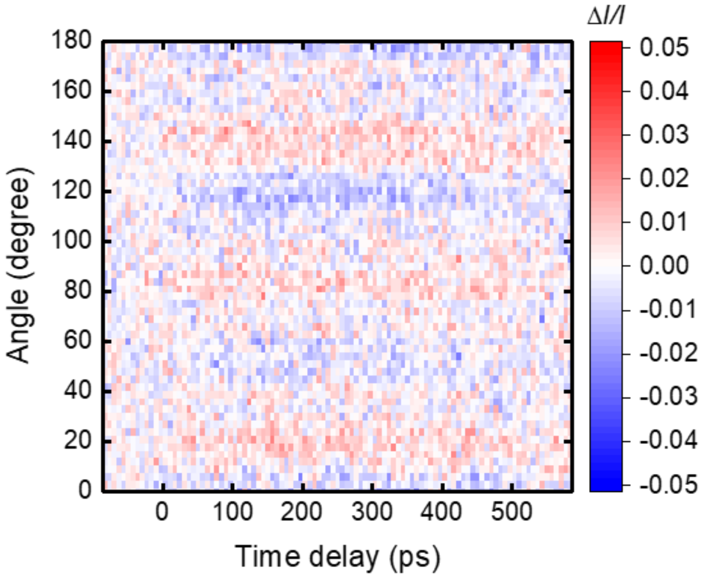


**Supplementary Figure 18.** Two-dimensional map of the angular- and time-dependent diffraction intensities (Supplementary Fig. 17) at the $Q$-value of 1.4 Å$^{-1}$. The map shows neither constructive nor destructive ordering due to signal accumulation[3]. In this case, the system contains a significant number of *cis*-azobenzene molecules before photoexcitation. The higher orientation of the azobenzene molecules in the LC phase occurs because a small number of bent molecules emerge among the majority of straight molecules. If *cis*-azobenzene accumulated, this effect would not be observed, suggesting that the isomerized molecules relax back to their initial structure in 5 ms (200 Hz), but they remain isomerized for 1 ms (1 kHz). Thus, the reverse reaction occurs in a few milliseconds.

**Supplementary Note 2**

The direction in which the molecules align is determined by the direction of the laser polarization axis; however, the rotational direction in which the molecules align is determined by both the direction of the laser polarization axis and the initial six-fold symmetric coordination of the azobenzene molecules. The laser polarization axis is fixed in the ultrafast time-resolved electron diffraction experiments. However, the initial six-fold symmetric coordination is random because the sample is fabricated via spin-coating. Therefore, we can observe both clockwise and counterclockwise rotations (Supplementary Fig. 19 and 20). If the six-fold symmetric coordination is initially aligned to the laser polarization axis, rotation does not occur (Supplementary Fig. 21).

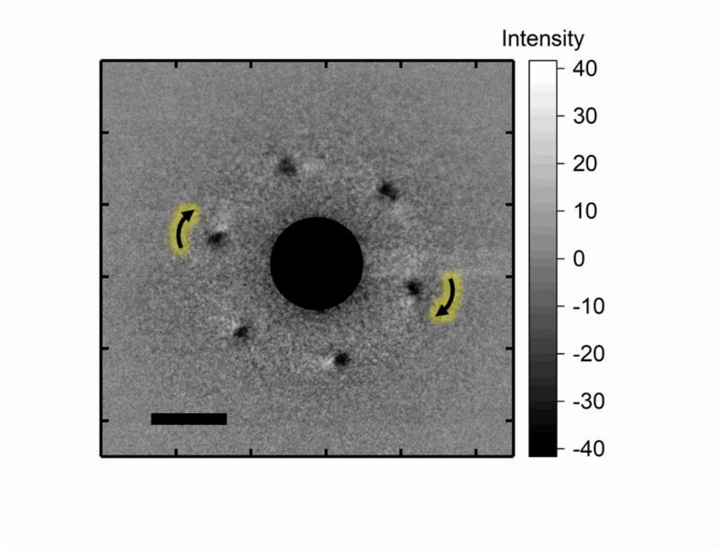


**Supplementary Figure 19.** Differential electron diffraction pattern of azobenzene molecules in the LC phase upon photoexcitation with linearly polarized UV light. The system rotates clockwise. The inset black scale bar corresponds to $Q = 1$ Å$^{-1}$.

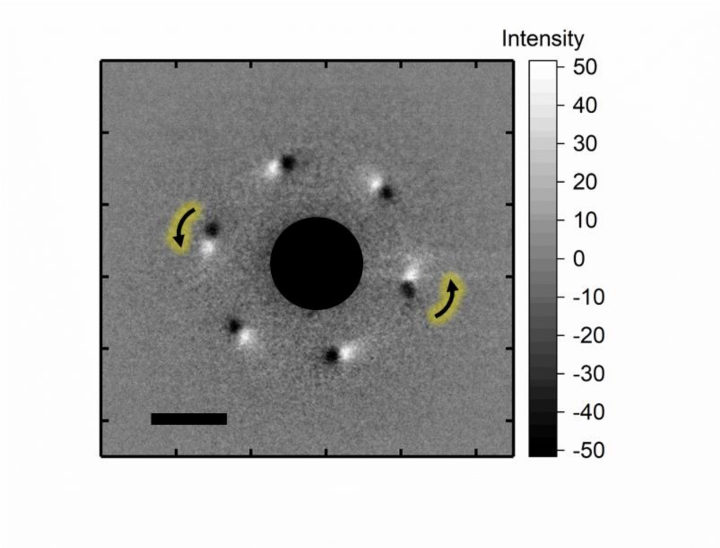


**Supplementary Figure 20.** Differential electron diffraction pattern of azobenzene molecules in the LC phase upon photoexcitation with linearly polarized UV light. The system rotates counter-clockwise. The inset black scale bar corresponds to $Q = 1$ Å$^{-1}$.

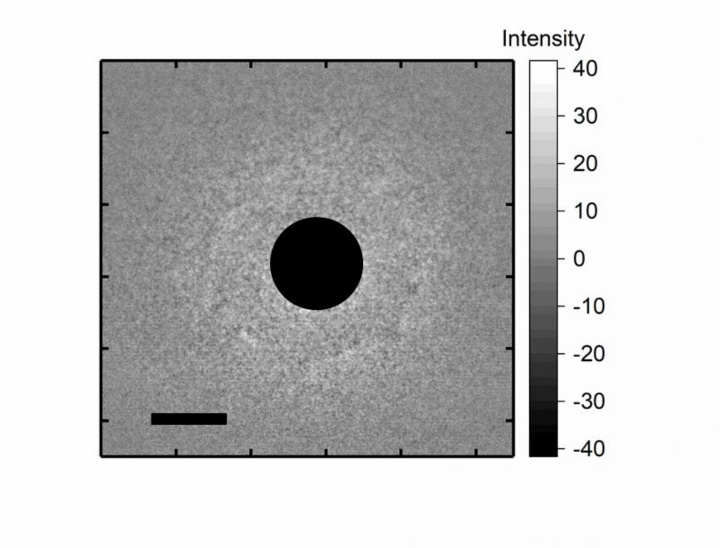


**Supplementary Figure 21.** Differential electron diffraction pattern of azobenzene molecules in the LC phase upon photoexcitation with linearly polarized UV light. No system rotation appears. The inset black scale bar corresponds to $Q$ = 1 $Å^{-1}$.

## 3. Molecular dynamics simulations

### Supplementary Note 3

Two-dimensional molecular dynamics (MD) calculations were performed based on the LAMMPS Molecular Dynamics Simulator[4]. As indicated in Ref. 5, the correlation among the interlayer molecules is much weaker than that among the intralayer molecules in the smectic B (Hex) phase; therefore, two-dimensional MD simulation should be appropriate. Azobenzene molecules were simplified as model spheroids, and the ratios of the three axes of the simplified *trans*- and *cis*-azobenzene molecules were 1:1:10 and 1:3:8, respectively (Supplementary Fig. 22). The potential energies of the azobenzene molecules were generated from the standard Gay-Berne model[6,7]. The normalized density of the molecules was used as a parameter (0.96, 0.97, and 0.98), and the density of the close-packed condition was set to 1.00. One hundred azobenzene molecules are contained in a unit system, and the photoexcitation can change an azobenzene molecule from the straight form to the bent form (1%). The timescale of the MD calculation is represented by the Lennard-Jones time unit (LJt). One period when a molecule vibrates on the Lennard-Jones potential corresponds to 1 LJt (a few picoseconds for soft matter). The azobenzene molecules in the LC phase before and after photoexcitation are represented by the systems without and with a bent molecule (Supplementary Figs. 23, 25, and 27), respectively. Supplementary Figs. 24, 26, and 28 show 20 calculated electron diffraction patterns (on the timescale of 5–100 LJt) at densities of 0.96, 0.97, and 0.98, respectively.

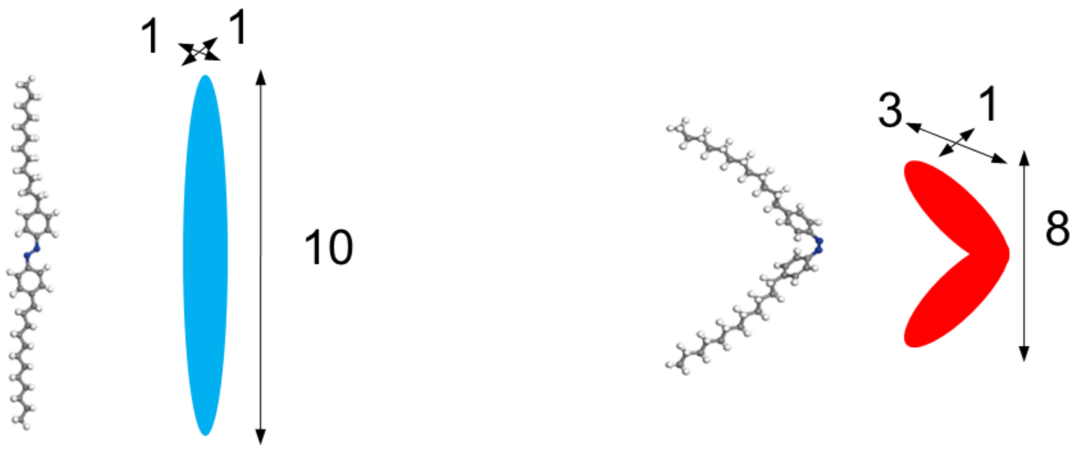


**Supplementary Figure 22.** Shapes of simplified azobenzene molecules.

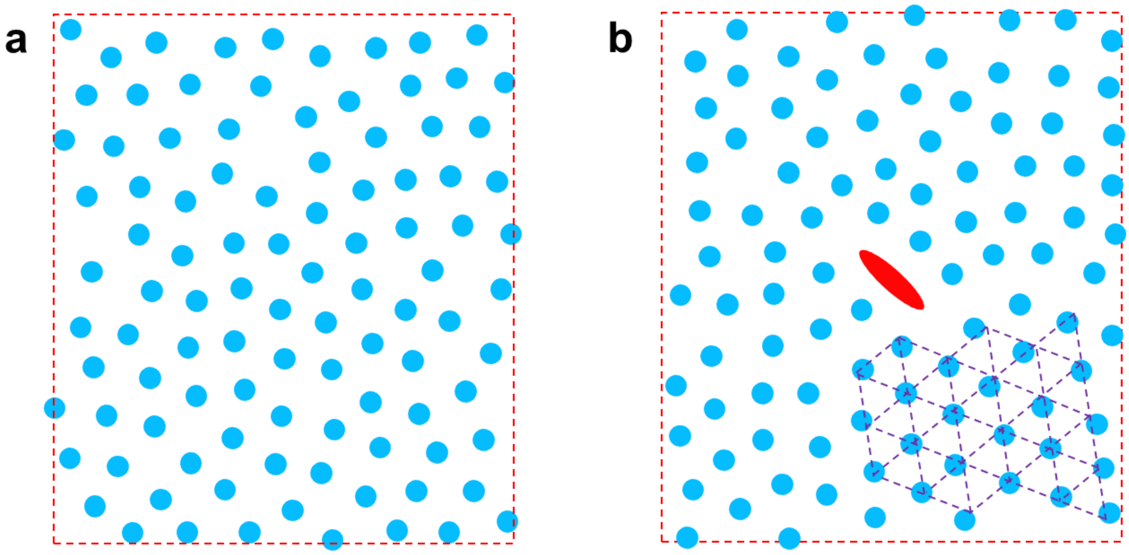


**Supplementary Figure 23.** Top views of typical molecular coordination without (a) and with (b) a bent molecule at a density of 0.96. One hundred molecules are contained in a unit of periodicity indicated by the red dashed boxes. The blue circles and the red ellipsoid represent straight and bent molecules, respectively. The purple dashed lines show the molecules aligned with six-fold symmetric coordination.

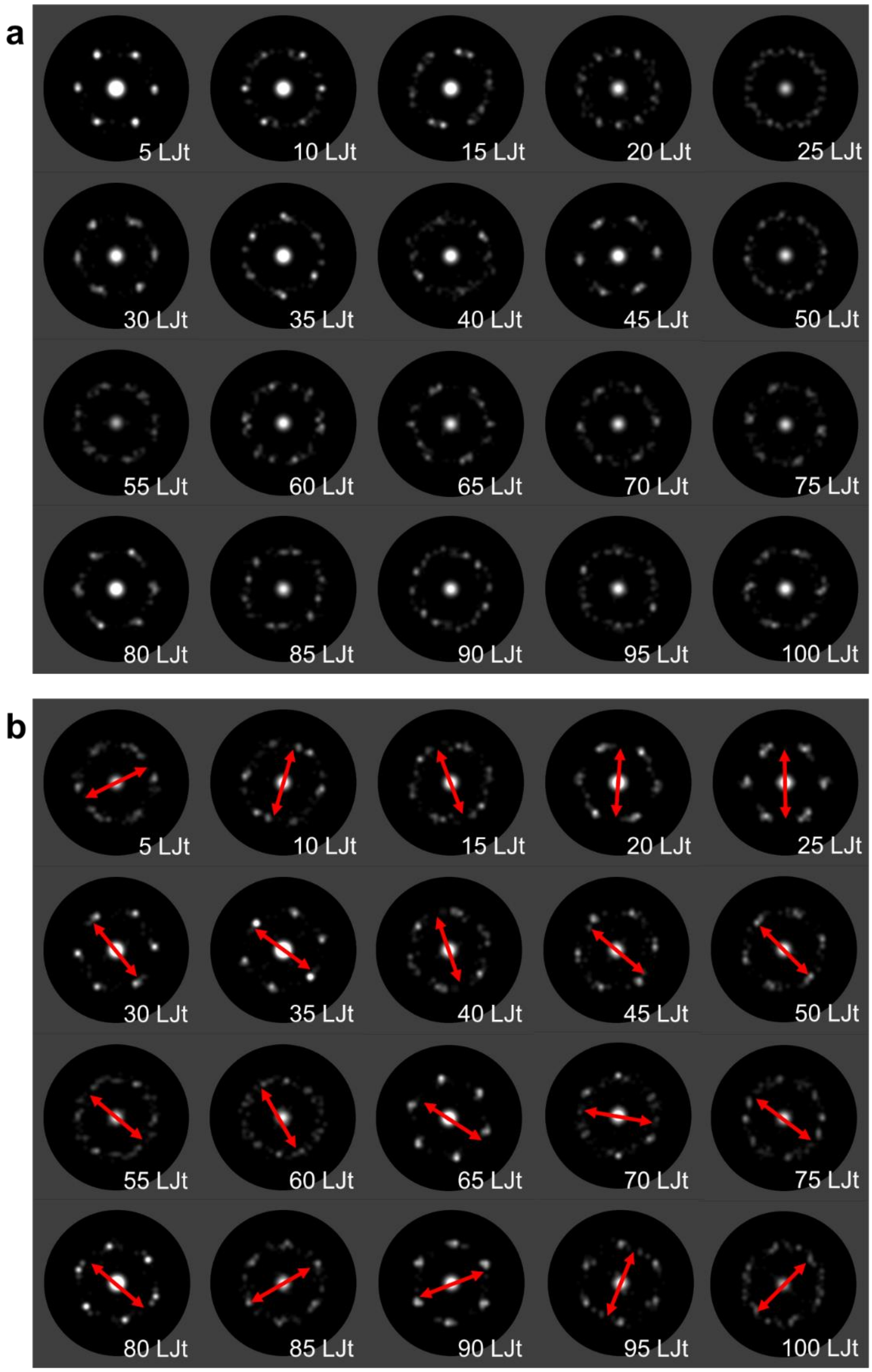


**Supplementary Figure 24.** Calculated diffraction patterns from the molecular coordinates calculated with the same conditions as in Supplementary Fig. 23a (a) and Fig. 23b (b). Red arrows in b indicate the direction of the bent molecules.

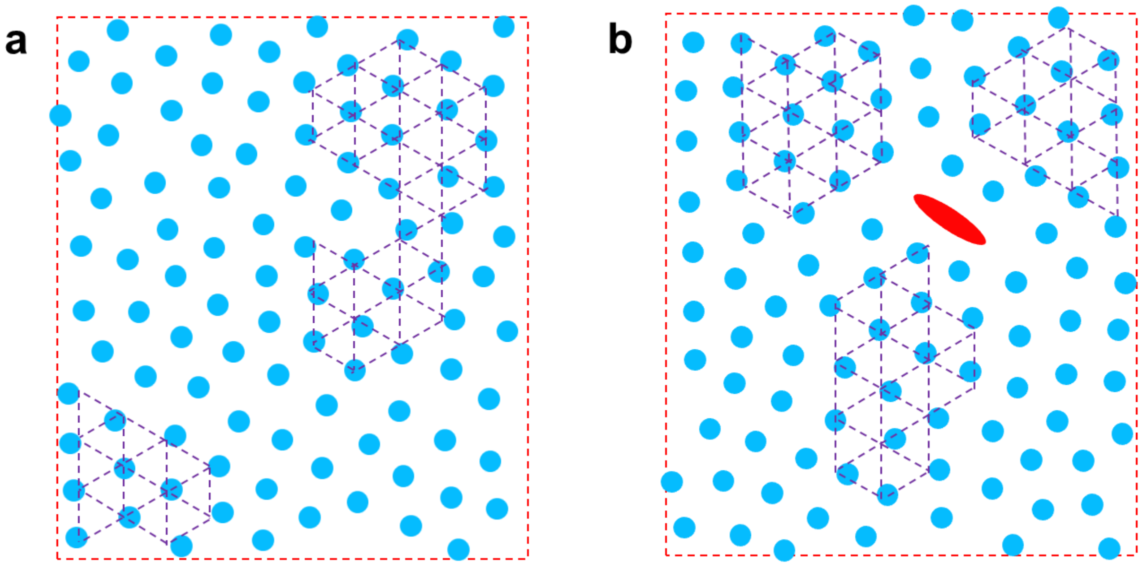


**Supplementary Figure 25**. Top views of typical molecular coordinates without (a) and with (b) a bent molecule at a density of 0.97.

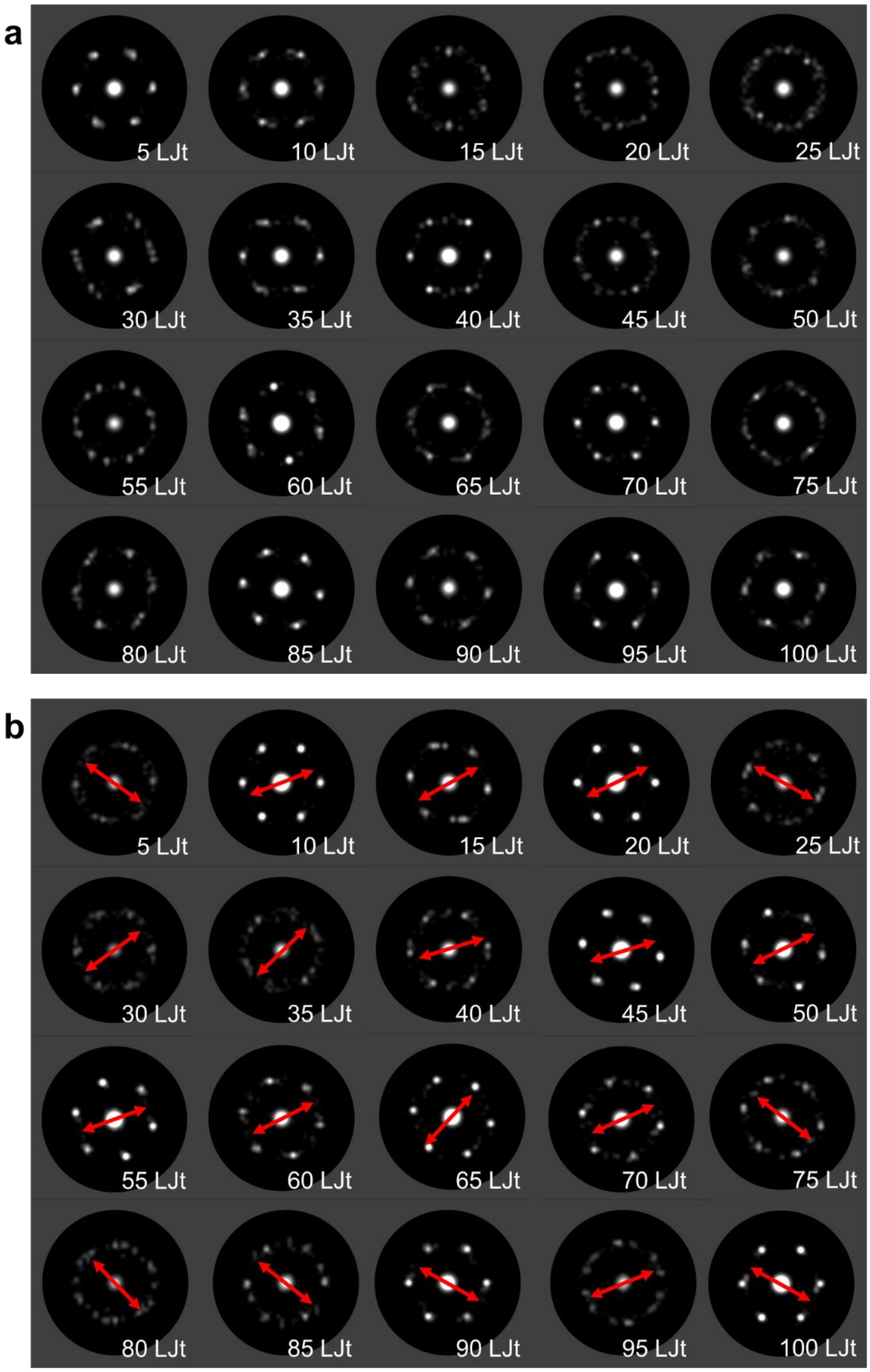


**Supplementary Figure 26.** Calculated diffraction patterns from the molecular coordinates calculated with the same conditions as in Supplementary Fig. 25a (a) and Supplementary Fig. 25b (b). Red arrows in b indicate the direction of the bent molecules.

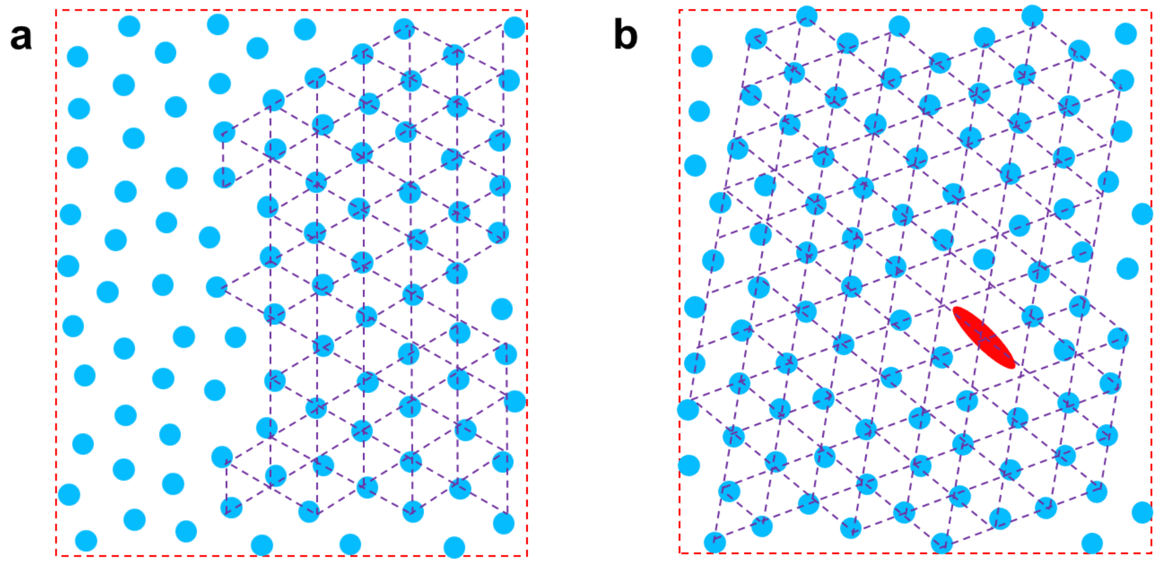


**Supplementary Figure 27.** Top views of typical molecular coordinates without (a) and with (b) a bent molecule at a density of 0.98.

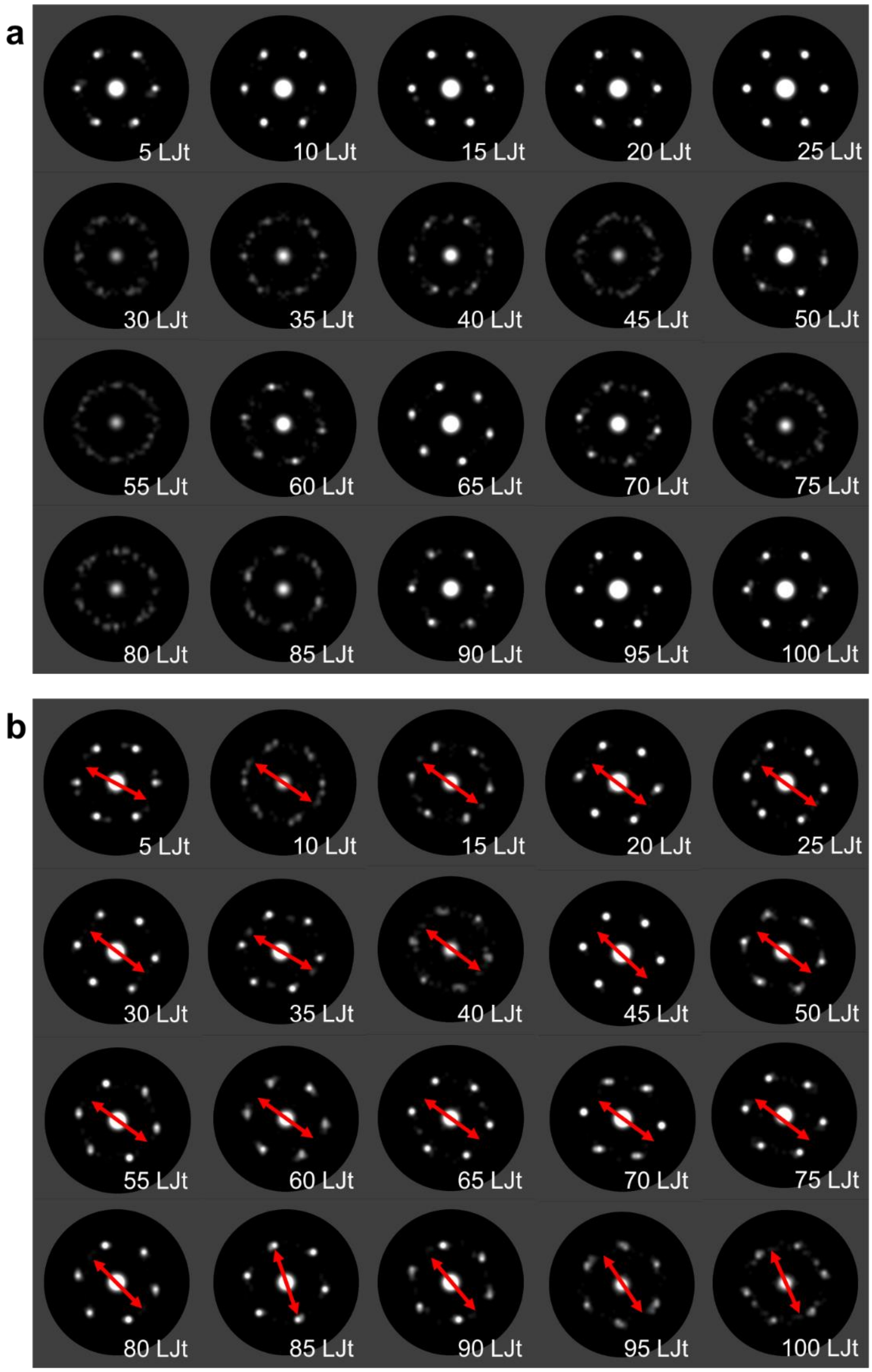


**Supplementary Figure 28.** Calculated diffraction patterns from the molecular coordinates calculated with the same conditions as in Supplementary Fig. 27a (a) and Supplementary Fig. 27b (b). Red arrows in b indicate the direction of the bent molecules.

**Supplementary Note 4**

Supplementary Figs. 23–28 show the higher orientation of the azobenzene molecules in the LC phase based on visual inspection. To statistically analyse the molecular coordinates obtained by the MD simulation, we defined the bond-order parameter[8] as $\varphi(\eta) = |\langle \exp(\mathrm{i}\eta\theta) \rangle|^2$, where $\eta$ and $\theta$ are the symmetry of the system and angle among three arbitrary molecules, respectively (Supplementary Fig. 29). As shown in Supplementary Fig. 30, the ideal hexagonal lattice represents a single peak in $\varphi(\eta)$ at $\eta = 6$ (six-fold symmetry). Supplementary Fig. 31 shows the bond-order parameters with and without a bent molecule from MD simulations (density: 0.98). As shown in the figures, the bond-order parameter with a bent molecule is approximately 50% higher than that without a bent molecule. This tendency agrees with the time-resolved electron diffraction measurements, i.e., the intensity of the electron diffraction spots increases by ~12% by photoexcitation.

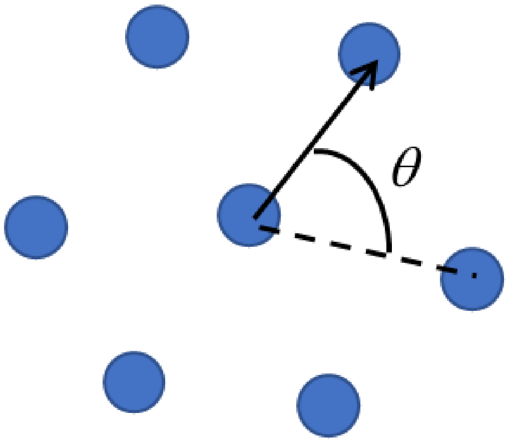


**Supplementary Figure 29.** The coordinates of the simplified molecules.

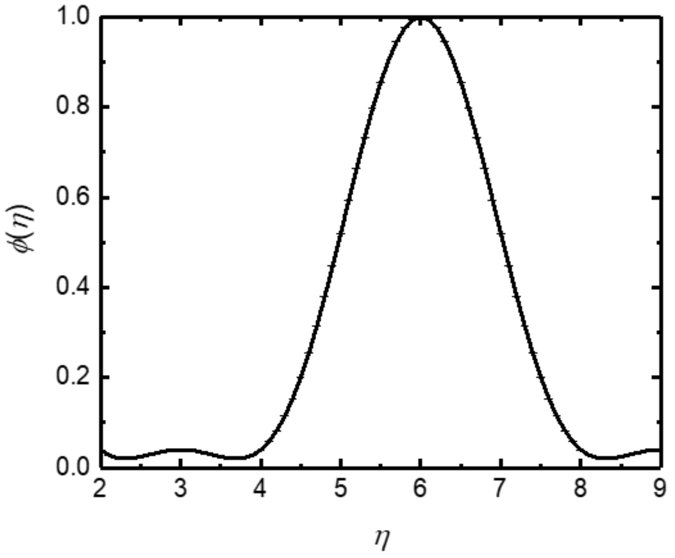


**Supplementary Figure 30.** The bond-order parameter of the ideal hexagonal lattice. The error bars represent the statistical error (standard error of the mean) at each $\eta$.

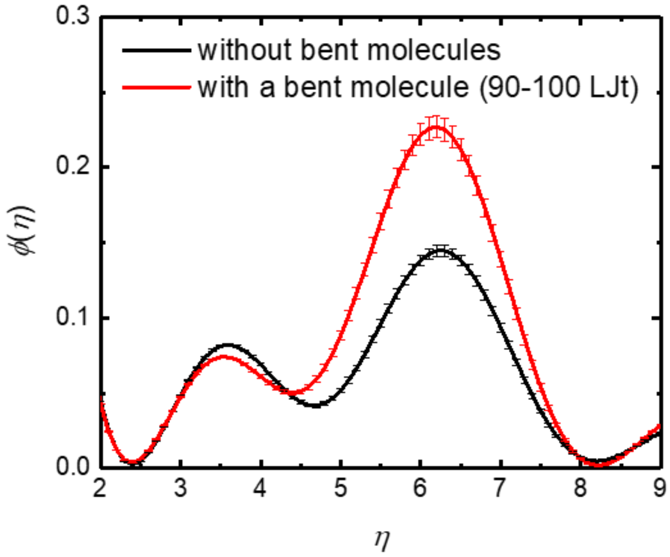


**Supplementary Figure 31.** The bond-order parameter of the molecular coordinates obtained from MD simulation (without bent molecules and with a bent molecule). The error bars represent the statistical error (standard error of the mean) at each $\eta$.

## Supplementary References


1. Moriyama, M., Song, S., Matsuda, H. & Tamaoki, N. Effects of doped dialkylazobenzenes on helical pitch of cholesteric liquid crystal with medium molecular weight: utilisation for fullcolour image recording. *J. Mater. Chem.* **11**, 1003–1010 (2001).
2. Hada, M. *et al.* Evaluation of Damage Layer in an Organic Film with Irradiation of Energetic Ion Beams. *Jpn. J. Appl. Phys.* **49**, 036503 (2010).
3. Gao, M. *et al.* Mapping molecular motions leading to charge delocalization with ultrabright electrons. *Nature* **496**, 343-346 (2013).
4. Plimpton, S. Fast parallel algorisms for short-range molecular dynamics. *J. Comp. Phys.* **117**, 1–19 (1995).
5. Goodby, J. W. et al. (eds) *Handbook of Liquid Crystals*, 2nd edn (Wiley-VCH, Weinheim, Germany, 2014).
6. Gay, J. G. & Berne, B. J., Modification of the overlap potential to mimic a linear site–site potential. *J. Chem. Phys.* **74**, 3316–3319 (1981).
7. Berardi, R., Muccioli, L., Orlandi, S., Ricci, M. & Zannoni, C. Computer simulations of biaxial nematics. *J. Phys.: Condens. Matter* **20**, 463101–463116 (2008).
8. Chaikin, P. M. & Lubensky, T. C. *Principles of condensed matter physics* (Cambridge Univ. Press, Cambridge, U. K., 1995).
